# HyperCube: A Small Lensless Position Sensing Device for the Tracking of Flickering Infrared LEDs

**DOI:** 10.3390/s150716484

**Published:** 2015-07-08

**Authors:** Thibaut Raharijaona, Paul Mignon, Raphaël Juston, Lubin Kerhuel, Stéphane Viollet

**Affiliations:** Aix-Marseille Université, ISM UMR 7287, 13288, Marseille Cedex 09, France; E-Mails: paul.mignon90@gmail.com (P.M.); rjuston@avenisense.com (R.J.); lubinkerhuel@yahoo.fr (L.K.); stephane.viollet@univ-amu.fr (S.V.)

**Keywords:** infrared sensing, visual motion, photodetector, motion tracking, mobile robots

## Abstract

An innovative insect-based visual sensor is designed to perform active marker tracking. Without any optics and a field-of-view of about 60°, a novel miniature visual sensor is able to locate flickering markers (LEDs) with an accuracy much greater than the one dictated by the pixel pitch. With a size of only 1 cm^3^ and a mass of only 0.33 g, the lensless sensor, called HyperCube, is dedicated to 3D motion tracking and fits perfectly with the drastic constraints imposed by micro-aerial vehicles. Only three photosensors are placed on each side of the cubic configuration of the sensing device, making this sensor very inexpensive and light. HyperCube provides the azimuth and elevation of infrared LEDs flickering at a high frequency (>1 kHz) with a precision of 0.5°. The minimalistic design in terms of small size, low mass and low power consumption of this visual sensor makes it suitable for many applications in the field of the cooperative flight of unmanned aerial vehicles and, more generally, robotic applications requiring active beacons. Experimental results show that HyperCube provides useful angular measurements that can be used to estimate the relative position between the sensor and the flickering infrared markers.

## 1. Introduction

Micro-aerial vehicles (MAVs) have required the development of very light-weight and miniature sensing devices. In [1], passive markers or light-emitting diodes (LEDs) are used in the visible spectrum. Therefore, cluttered environments and low-light conditions make their performance decrease. The design of advanced miniature sensors that can substitute for classical devices, such as bulky stereoscopic cameras [2] or ultrasonic sensors [3], is challenging. In [4], ultrasonic systems have been reviewed in detail. Their use in location estimation is discussed in terms of performance, accuracy and limitations. In [5], an acoustic location system for indoor applications is presented. It estimates the target position based on a fingerprinting technique. The result is similar to the Wi-Fi radio-based localization system. Recently, a novel class of optical sensors has been introduced for aerial applications. For instance, new flexible compound-eyes have been proposed for motion extraction and proximity estimation based on the optic flow principle [6,7]. Censi *et al.* [8] present an approach to low-latency pose tracking using an event-based camera in [9] with active LED markers, which are infrared (IR) LEDs blinking at a high frequency (>1 kHz). The advantage of this camera is that it can track frequencies up to several kilohertz. Monocular vision-based pose estimation is proposed, while accuracy, versatility and performance are improved. Pose estimation is performed with very low latencies. Nevertheless, the precision is constrained by the low sensor resolution, which is 128 × 128 pixels. It is not a commercial technology, and it requires specific skills in programming event-based sensors. In [10], an accurate, efficient and robust pose estimation system based on IR LEDs is also proposed. The system is used to stabilize a quadrotor, both indoors and outdoors. Furthermore, a proximity sensor has also been presented in [11,12]. These studies depict an embedded IR-based 3D relative positioning sensor dedicated to inter-robot spatial-coordination and cooperative flight. Dealing with the ability to determine the spatial orientation and placement of the unmanned aerial platform in real time, the cooperative flight tends to incorporate leader-follower UAVs using vision processing, radio-frequency data transmission and more sensors. Then, the cooperative flight requires the control of only one UAV and should allow the deployment of multiple UAVs. Etter *et al.* [13] have designed and evaluated a leader-follower autonomous aerial quadrotor system for search and rescue operations using an IR camera and multiple IR beacons. The system incorporates vision processing, radio-frequency data transmission and additional sensors to achieve flocking behavior. Thanks to numerical simulations, IR beacons installed on the platform provides accurate data with respect the spatial orientation and the placement of the system units. Nevertheless, in sunny conditions or with other infrared sources, the IR camera used will have noisy signals. Masselli *et al.* [14] have proposed a novel method for pose estimation for MAVs using passive visual markers and a monocular color camera mounted onto the MAV as the only sensor. For pose estimation, the P3Pproblem is solved in real time onboard the MAV. The perspective aims at filtering the pose estimate with a Kalman filter in order to accelerate the overall method.

**Figure 1:**
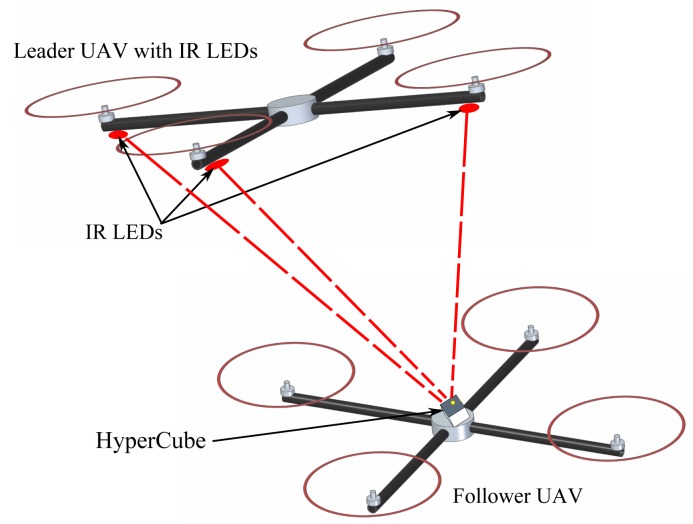
Sketch of a collaborative flight using IR LEDs and the HyperCube sensor embedded onboard the follower.

In this paper, we present a novel miniature optical sensor without any optics, called HyperCube, endowed with hyperacuity [15] for active IR LED marker tracking applications. The principle on which HyperCube is based was developed by Kerhuel *et al.* and detailed in [16,17]. In [16], hyperacuity was obtained at a very low cost. The Vibrating Optical Device for the Kontrol of Autonomous robots (VODKA) sensor was mounted onto a miniature aerial robot that was able to track a moving target accurately by exploiting the robot's uncontrolled random vibration as the source of its microscanning movement. In [17], an improved bio-inspired optical position sensing device was designed. Insect-based retinal microscanning movements were used to detect and locate contrasting objects, such as edges or bars. The active micro-vibrations imposed upon the retina endowed the sensor with hyperacuity. In this new paper, we designed a novel miniature bioinspired sensor where the mechanical vibration is replaced by a flickering LED. It is demonstrated that using only three photosensors with a sensitive area equal to 0.23 mm^2^, we obtained hyperacuity by replacing the micro-movements with a modulation of infrared LEDs. The markers in use are off-the-shelf IR LEDs flickering at a high frequency (>1 kHz). We envision that this sensor could be used for cooperative flight, as presented in Figure 1.

The purpose of this paper is to present an alternative and a minimalistic solution involving three pixels for 3D localization with respect to IR markers. HyperCube assesses azimuth and elevation measurements of a pattern (planar object) composed of flickering IR LEDs. We also show the abilities of HyperCube to measure its relative position with respect to the markers. We propose a pose estimation system that consists of multiple flickering IR LEDs and a lightweight optical sensor. The LEDs are attached to a moving target, and the sensor provides angular measurements. We also show that the pose estimation is computationally inexpensive.

The paper is organized as follows: Section 2 details the principle of the sensor, the visual processing algorithm and its advantages. The principle of the sensor is discussed in Section 3. The experimental setup is presented in Section 4. Section 5 shows the experimental results.

## 2. Modeling the HyperCube Sensor

The prototype was built using off-the-shelf elements. Its volume is 1 cm^3^ and it weights 0.33 g. These characteristics make it inexpensive, light, small and power-lean.

### 2.1. Angular Sensitivity of the Photosensors

**Figure 2:**
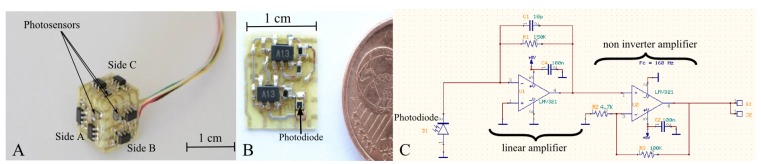
(**A**) Each side of the HyperCube sensor integrates one photosensor and an analog amplifier for the conversion of the photodiode current into an output voltage; (**B**) one side of the HyperCube sensor with one photosensor (tiny surface-mounted device (SMD) photodiode); (**C**) electrical schematic drawing of the photoconductor circuit.

The HyperCube sensor is composed of three surface-mounted device (SMD) photodiodes (VISHAY TEMD7000). Each photodiode (or photosensor) is located on each side of the cube, as shown in Figure 2A. Thanks to this cubic assembly, the optical axes of each pair of photosensors are separated by an angle Δ*φ* = 90°. On each side of the sensor, an analog circuit (Figure 2B) converts the output current of the photodiode into a proportional voltage thanks to a photoconductor electronic circuit (Figure 2C). The linear amplifier in Figure 2C is an analog low-pass filter with a cutoff frequency equal to 106 kHz. Considering the high gain given by the resistance of 150 kΩ, the linear amplifier aims at preventing the signal from oscillations.

HyperCube can extract two angular positions (azimuth and elevation) from the position of an object composed of several IR LEDs. The IR LEDs used in the experimental setup are the OSRAM SFH4232. Their spectrum has a maximum emissive power at a wavelength of 900 nm, which corresponds to the maximum of absorption of the photosensors. Thereafter, the IR LEDs will be considered as a point source. The signal processing used to estimate the elevation and azimuthal angles from the three HyperCube output signals runs in real time onboard a custom-made electronic board described in Section 2.2.

### 2.2. Model of the Photosensor Output Signal

**Figure 3:**
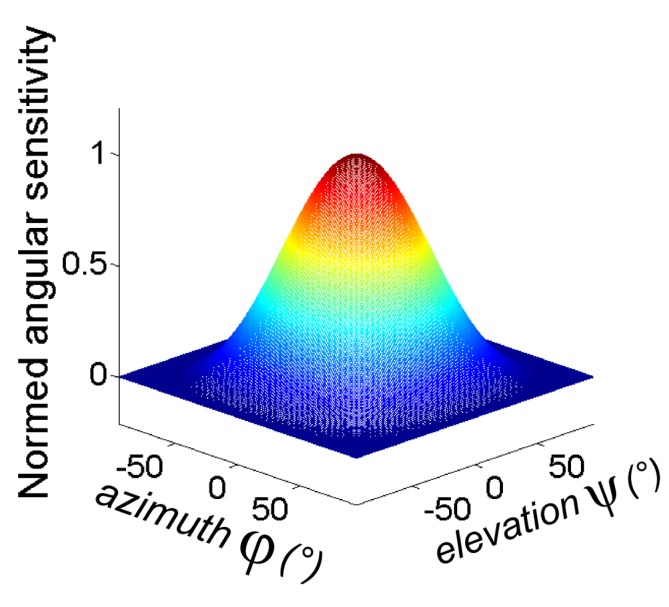
Cosine-like angular sensitivity of a photosensor.

The cosine-like angular sensitivity function for each photosensor depends on the azimuth and elevation, as shown in Figure 3.

**Figure 4:**
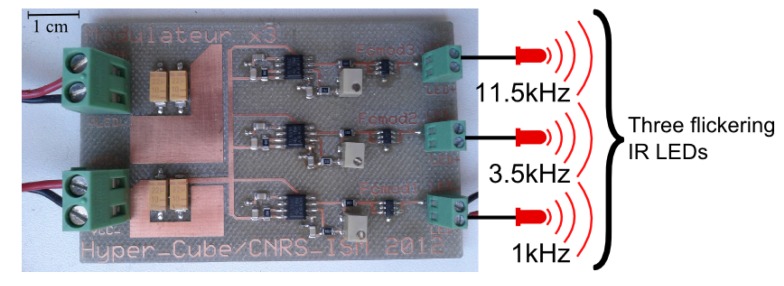
Custom-made electronic board for the frequency modulation of the IR LEDs. It produces three separate signals that flicker at 1 kHz, 3.5 kHz and 11.5 kHz, sent to the three IR LEDs of the object to be located.

**Figure 5:**
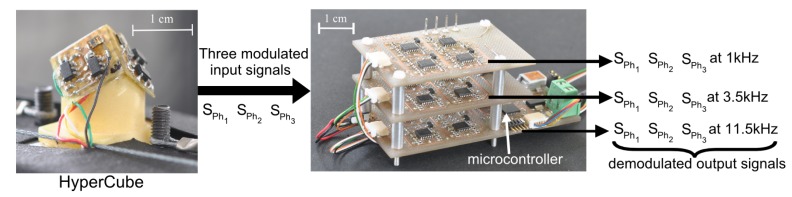
Custom-made acquisition and demodulation board. It is composed of three analog demodulation circuits. A microcontroller processes the visual output signal yielded by HyperCube and provides the azimuth and the elevation, which are sent through a USB interface to a PC.

Each IR LED signal is modulated at a specific frequency *f_i_*. The modulation is provided by a custom-made electronic board (Figure 4). The latter was designed to provide one specific modulation frequency *f_i_* to each LED, such that *f*_1_ = 1 kHz, *f*_2_ = 3.5 kHz, *f*_3_ = 11.5 kHz for the three separate IR LED emitters of the pattern. Then, an additional custom-made processing board (Figure 5) achieved the analog signal demodulation for each frequency *f_i_*. To summarize, two electronic boards have been designed: the modulation of the LEDs is performed by the first board in Figure 4, and the second electronic board in Figure 5 performs the analog demodulation and the digital visual processing.

Once the three HyperCube output signals are demodulated, amplified and filtered by a low-pass filter at 100 Hz, one can approximate for each photosensor *Ph_i_* and frequency *f_i_*, the output signal *S_ph_i_,f_i__* of HyperCube.

A Gaussian-like directivity function mimics the Gaussian angular sensitivity function of flies' photoreceptors [18,19]. The Gaussian-like angular sensitivity of each photosensor can be defined by the angle of acceptance, denoted Δ*ρ*. The angle Δ*ρ* is defined as the full width at half maximum, as depicted later in Figure 9. The Gaussian-like angular sensitivity of a photosensor is given by:

(1) $$\sigma \left(\theta \right)=\frac{2\sqrt{\pi \text{ln}\left(2\right)}}{\pi \Delta \rho }e^{-4\text{ln}\left(2\right)\left(\frac{\theta^{2}}{\Delta \rho^{2}}\right)}$$

where *θ* is the angle between the photosensor's optical axis and that of a point light source. We also introduce the cosine-like angular sensitivity function *σ*, such that:

(2) $$\sigma \left(\theta \right)=\{\begin{array}{cc} \cos\left(\theta \right) & if\left|\theta \right|\leq \frac{\pi }{2}\\ 0 & \text{else} \end{array}$$

**Figure 6:**
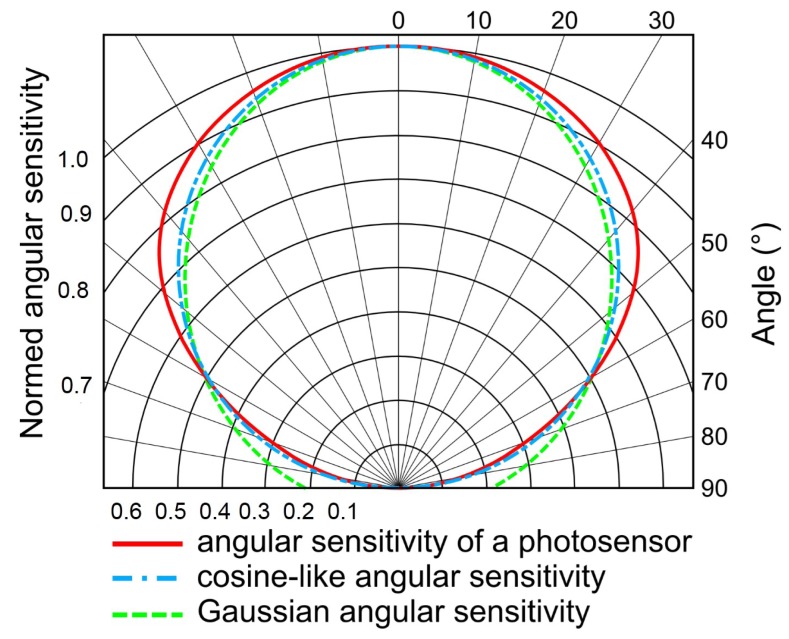
Comparison between the angular sensitivity of a photosensor embedded on HyperCube (red solid line) and the cosine-like angular sensitivity of the model (blue dashed dot line) given by Equation (2) and the Gaussian angular sensitivity (dashed green line) given by Equation (1) with the acceptance angle Δ*ρ* = 120°.

From Figure 6, the angular sensitivity of a photosensor as a blue dotted dashed line is compared to the Gaussian angular sensitivity given in Equation (1) as a green dashed line. One can note that the angular sensitivity of a photosensor does not strictly shape the Gaussian-like one and could also be approximated by a cosine-like angular sensitivity, which is plotted in red.

**Figure 7:**
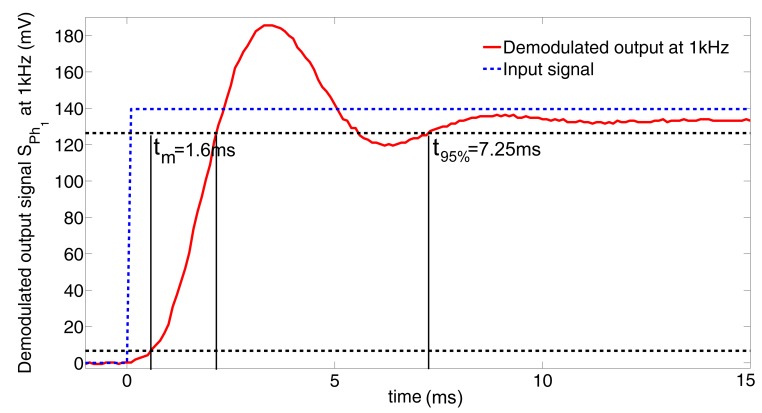
Experimental recording of the dynamic response of the demodulated output signal *S_Ph_*_1_ at 1 kHz. The settling time at 95% *t*_95%_ is equal to 7.25 ms, and the rise time *t_m_* (the time required for the response to rise from 5% to 95% of its final value) is equal to 1.6 ms.

To characterize the dynamic response of the HyperCube sensor, we measured the demodulated output signal of a photosensor at 1 kHz in response to a step input. We turned on a modulated IR LED placed in front of the photosensor *Ph*_1_, and we measured the demodulated output signal by means of the board, as shown in Figure 5. To precisely know the step time, the power input of the modulation electronic board (+3.3 V) is connected to a digital input of the microcontroller. The sampling frequency is 10 kHz. As depicted in Figure 7, the settling time *t*_95%_ is equal to 7.25 ms, and the rise time *t_m_* is equal to 1.6 ms. Therefore, given these dynamic properties, the use of the HyperCube sensor could be relevant for MAV applications.

**Figure 8:**
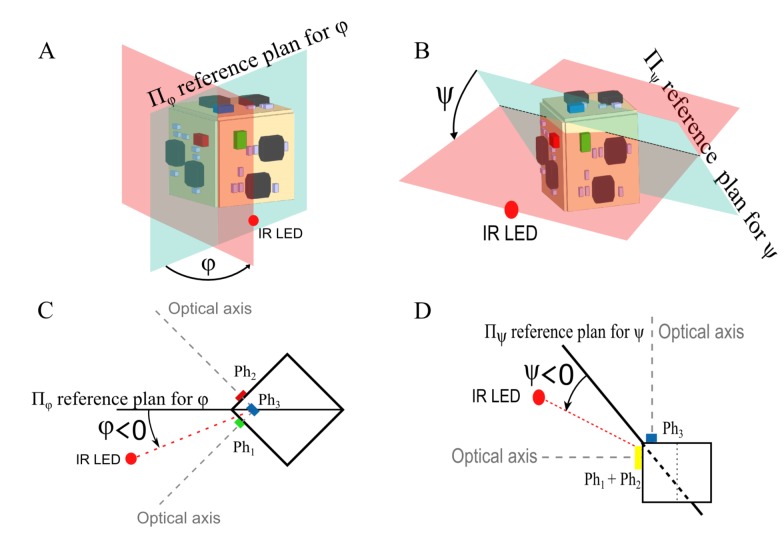
(**A**) Front view, which gives an illustration of the azimuth *φ* and the reference plane Π*_φ_*; (**B**) front view, which gives an illustration of the elevation angle *ψ* and the reference plane Π*_ψ_*; (**C**) top view with the reference plane Π*_φ_*, which is the plane through the IR LED and the optical axes of the photosensor *Ph*_1_ and the photosensor *Ph*_2_; (**D**) side view with the reference plane Π*_ψ_*, which is the plane through the IR LED and the optical axes of the virtual photosensor (*Ph*_1_ + *Ph*_2_) and the photoreceptor *Ph*_3_.

From Figure 8, the azimuth angle *φ* and the elevation angle *ψ* are defined.

Therefore, *S_Ph_i_,f_i__* the output signal of the photodiode *i* is related to a specific flickering frequency *f_i_* as follows:

(3) $$\begin{array}{c} S_{Ph_{1},f_{i}}\left(\varphi ,\psi \right)=A_{1,i}.\sigma \left(\varphi -\frac{\pi }{4}\right).\sigma \left(\psi +\frac{\pi }{4}\right)\\ \text{if}\left|\varphi -\frac{\pi }{4}\right|<\frac{\pi }{2}\;\text{and}\;\left|\psi +\frac{\pi }{4}\right|<\frac{\pi }{2}\;\text{else}\;S_{Ph_{1},f_{i}}\left(\varphi ,\psi \right)=0 \end{array}$$

(4) $$\begin{array}{c} S_{Ph_{2},f_{i}}\left(\varphi ,\psi \right)=A_{2,i}.\sigma \left(\varphi +\frac{\pi }{4}\right).\sigma \left(\psi +\frac{\pi }{4}\right) \end{array}$$

(5) $$\begin{array}{c} \text{if}\left|\varphi +\frac{\pi }{4}\right|<\frac{\pi }{2}\;\text{and}\;\left|\psi +\frac{\pi }{4}\right|<\frac{\pi }{2}\;\text{else}\;S_{Ph_{2},f_{i}}\left(\varphi ,\psi \right)=0\\ S_{Ph_{3},f_{i}}\left(\varphi ,\psi \right)=A_{3,i}.\sigma \left(\varphi \right).\sigma \left(\psi +\frac{\pi }{4}\right)\\ \text{if}\left|\varphi \right|<\frac{\pi }{2}\;\text{and}\;\left|\psi -\frac{\pi }{4}\right|<\frac{\pi }{2}\;\text{else}\;S_{Ph_{3},f_{i}}\left(\varphi ,\psi \right)=0 \end{array}$$

With *A*_1,_*_i_, A*_2,_*_i_, A*_3,_*_i_*, the gains of the photosensors *Ph*_1_, *Ph*_2_ or *Ph*_3_ on each side of the HyperCube sensor. The index *i* refers to the frequencies 1 kHz, 3.5 kHz, 11.5 kHz. *φ* and *ψ* are the azimuth and elevation with respect the IR LED *i*.

**Figure 9:**
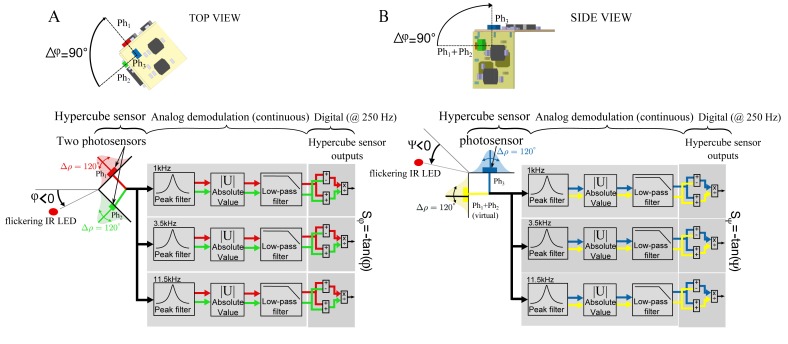
Sketch diagram of the HyperCube signal processing algorithm. (**A**) Top view: the sensor measures the azimuth *φ*. The left part shows the IR LED modulated at a frequency noted *f_i_* (1 kHz, 3.5 kHz or 11.5 kHz). In this view, HyperCube is composed of two photosensors *Ph*_1_ and *Ph*_2_ with their respective cosine-like angular sensitivities corresponding to Sides A and C of the sensor (see Figure 2A). An analog band-pass filter acts as a demodulator to extract the signal corresponding to the frequency *f_i_* of the IR LED, and an analog low-pass filter section with the cut-off frequency of 100 Hz reduces the high-frequency noise and prevents the subsequent analog-to-digital conversion from any aliasing effects. The digital processing consists of computing for each frequency *f_i_* the ratio of the relative difference to the sum between the two signals $\left(\frac{S_{Ph_{1}}-S_{Ph2}}{S_{Ph_{1}}+S_{Ph_{2}}}\right)$ to yield the HyperCube sensor output signal; (**B**) Side view: the same signal processing is applied on the signal provided by the photosensor *Ph*_3_ (Side B in Figure 2A) of HyperCube and a virtual photosensor, which is the sum of the photosensors *Ph*_1_ and *Ph*_2_.

The three photosensor (photodiode) output signals are processed by a low-cost microcontroller from Microchip (dsPIC33FJ128GP802). As shown in Figure 9A,B, the digital processing operates at 250 Hz and computes the relative difference over the sum of two adjacent photosensor signals. For each frequency 1 kHz, 3.5 kHz and 11.5 kHz, respectively, a custom-made demodulation board was designed as depicted in Figure 5. Therefore, for each frequency *f*_i_, a Sallen Key peak filter and a low pass filter with a cut off frequency equal to 100 Hz were chosen. The demodulated output signals , *S*_*Ph*_2__ and *S*_*Ph*_3__ were processed at 1kHz, 3.5 kHz and 11.5 kHz, respectively.

### 2.3. Advantages of HyperCube

HyperCube exhibits several advantages over other conventional sensors, like CMOS cameras, according to these following points:

- low cost: there is no optics; the angular position measurement relies only on the angular sensitivity of photodiodes, which is, in essence, non-uniform (cosine law) and only requires very inexpensive off-the-shelf electronic components.
- low computational resources: the measurements are the angular information; this does not require any image processing; a high refresh rate for the the same computational resource can easily be reached.
- small size: because of a low number of components, the sensor is light weight (0.33 g) and very compact (1cm^3^).

## 3. Principle of the HyperCube Sensor

### 3.1. Angle Reconstruction

Let us consider the azimuth angle *φ*. It is defined as the angle between the reference plane Π*_φ_* (see Figure 8A) and a plane including the IR LED (see the red dotted line in Figure 8C). Π*_φ_* is the mid-plane between the photosensors *Ph*_1_ and *Ph*_2_. The second plane includes the IR LED and the intersection between the optical axes of *Ph*_1_ and *Ph*_2_. In Figure 8A, the azimuth *φ* is presented in front view and in top view in Figure 8C.

The digital processing operated in the microcontroller with respect to the azimuth *φ* returns an output signal whose theoretical expression can be written as:

(6) $$S_{\varphi }=\frac{S_{ph_{1}}-S_{ph2}}{S_{ph_{1}}+S_{ph_{2}}}$$

Equation (6) gives the output signal, which results from two photosensors (*Ph*_1_ and *Ph*_2_, respectively), to obtain the ratio between the difference and the sum of the differentiated and demodulated photosensor signals. This expression reduces the common mode noise signal introduced by the artificial light. Moreover, the expression of Equation (6) results from previous studies carried out at the laboratory on hyperacute visual sensors based on active micro-movements applied to an artificial eye in [16,17]. It is worth noting that we obtained here hyperacuity by replacing the micro-movements with a modulation of IR LEDs. From Equation (6) using Equations (3) and (4), one can write:

(7) $$S_{\varphi }=\frac{A_{1}\sigma \left(\varphi +\frac{\pi }{4}\right)\cdot \sigma \left(\psi +\frac{\pi }{4}\right)-A_{2}\sigma \left(\varphi -\frac{\pi }{4}\right)\cdot \sigma \left(\psi +\frac{\pi }{4}\right)}{A_{1}\sigma \left(\varphi +\frac{\pi }{4}\right)\cdot \sigma \left(\psi +\frac{\pi }{4}\right)+A_{2}\sigma \left(\varphi -\frac{\pi }{4}\right)\cdot \sigma \left(\psi +\frac{\pi }{4}\right)}$$

*A*_1_ and *A*_2_ are the amplitudes of the signals delivered by the photosensors *Ph*_1_ and *Ph*_2_. If we assume *A*_1_ = *A*_2_, Equation (7) gives:

(8) $$S_{\varphi }=\frac{\cos\left(\varphi +\frac{\pi }{4}\right)-\cos\left(\varphi -\frac{\pi }{4}\right)}{\cos\left(\varphi +\frac{\pi }{4}\right)+\cos\left(\varphi -\frac{\pi }{4}\right)}$$

Therefore:

(9) $$S_{\varphi }=-\tan\left(\varphi \right)$$

The elevation angle *ψ* is defined as the angle between the reference plane Π*_ψ_* (see Figure 8B) and a plane including the IR LED (see the red dotted line in Figure 8D). The second plane includes the IR LED and the intersection of the optical axes of the virtual photodiode (*Ph*_1_ + *Ph*_2_) and *Ph*_3_. In Figure 8B, the elevation angle is presented in front view and in side view in Figure 8D.

The digital processing with respect to the elevation angle *ψ* involves a virtual photosensor (*Ph*_1_ + *Ph*_2_), which is the combination of *Ph*_1_ and *Ph*_2_. In the following, we introduce the signal *S_Ph_virtual__* delivered by the virtual photosensor:

$$\begin{array}{c} S_{Ph_{\text{virtual}}}=S_{Ph_{1}}+S_{Ph_{2}}=A\sigma \left(\psi +\frac{\pi }{4}\right).\left[\sigma \left(\varphi +\frac{\pi }{4}\right)+\sigma \left(\varphi -\frac{\pi }{4}\right)\right]\\ S_{Ph_{\text{virtual}}}=\{\begin{array}{cc} A\sigma \left(\psi +\frac{\pi }{4}\right) & if\left|\varphi \right|<\frac{\pi }{4}\\ S_{Ph_{1}} & if\frac{\pi }{4}<\varphi <\frac{3\pi }{4}\\ S_{Ph_{2}} & if-\frac{3\pi }{4}<\varphi <-\frac{\pi }{4}\\ 0 & \text{else} \end{array} \end{array}$$

Similarly to Equation (9), if we assume the amplitudes are such that *A* = *A*_1_ = *A*_2_ = *A*_3_, the digital signal output for the elevation angle *ψ* gives: $S_{\psi }=\frac{S_{Ph_{\text{virtual}}}-S_{Ph3}}{S_{Ph_{\text{virtual}}}+S_{Ph3}}$. Using the definition of *S_Ph_virtual__*, one can write if $\left|\varphi \right|<\frac{\pi }{4}$:

(10) $$S_{\psi }=\frac{\sigma \left(\psi +\frac{\pi }{4}\right)-\sigma \left(\varphi \right).\sigma \left(\psi -\frac{\pi }{4}\right)}{\sigma \left(\psi +\frac{\pi }{4}\right)-\sigma \left(\varphi \right).\sigma \left(\psi -\frac{\pi }{4}\right)}$$

Considering the approximation of small angles for *φ, σ* (*φ*) ≃ 1, one can simplify:

$$S_{\psi }=\frac{\sigma \left(\psi +\frac{\pi }{4}\right)-\sigma \left(\psi -\frac{\pi }{4}\right)}{\sigma \left(\psi +\frac{\pi }{4}\right)+\sigma \left(\psi -\frac{\pi }{4}\right)}=\frac{\cos\left(\psi +\frac{\pi }{4}\right)-\cos\left(\psi -\frac{\pi }{4}\right)}{\cos\left(\psi +\frac{\pi }{4}\right)+\cos\left(\psi -\frac{\pi }{4}\right)}$$

Therefore:

(11) $$S_{\psi }≃-\tan\left(\psi \right)$$

### 3.2. Position Estimation

Given the signal outputs *S_ψ_* and *S_φ_* and one IR LED, the first step was to assess the relative position of the IR LED in the plane (*X_LED_,Y_LED_*) with respect to HyperCube. As described in the experimental setup presented in Section 4, if we assume *Ẑ_LED_* is *a priori* known or assessed, using only angular measurements provided by HyperCube, the relative position of the IR LED with respect the sensor can be estimated such as:

(12) $${\hat{X}}_{\text{LED}}=-\tan\left(\varphi \right){\hat{Z}}_{\text{LED}}$$

(13) $${\hat{Y}}_{\text{LED}}=-\tan\left(\psi \right){\hat{Z}}_{\text{LED}}$$

where *φ* < 0 and *ψ* < 0 as defined in Figure 9A,B.

The experimental setup detailed in Section 4 justifies the approximation of the small angles. Therefore, one can assume that:

(14) $${\hat{X}}_{\text{LED}}=-\varphi \cdot {\hat{Z}}_{\text{LED}}$$

(15) $${\hat{Y}}_{\text{LED}}=-\psi \cdot {\hat{Z}}_{\text{LED}}$$

## 4. Experimental Setup

**Figure 10:**
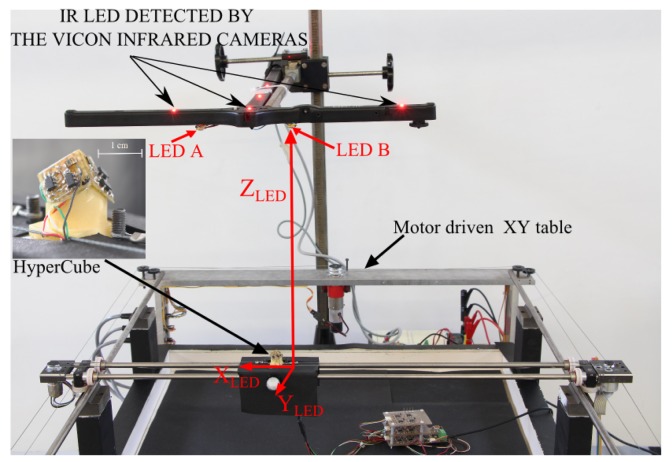
XY table driven by DC motors. The HyperCube position is controlled in closed-loop by means of two incremental encoders. The distance *Z_LED_* was assumed here to be known and constant. Additional active motion markers were used to compare the precision of HyperCube with the ground truth acquired from the VICON motion capture system.

In this work, we aimed at demonstrating that the estimation of the relative position of an IR LED with respect to HyperCube can be achieved by means of angular measurements. The experimental setup is composed of a motor-driven XY table, as shown in Figure 10. We oriented HyperCube so that it was pointing upward. HyperCube can move along the X and Y directions thanks to two DC motors.

Two incremental encoders measure the HyperCube position along the *X* and *Y* directions. In the initial conditions, which define the origin of the inertial frame, an IR LED is located just above HyperCube at a height *Z_LED_* = 300 mm. As an absolute sensor's position of reference, a motion capture system provided by the company VICON was used for measuring the ground truth, *i.e.*, the precise absolute position of HyperCube. The tracking system is equipped with 17 IR cameras and IR active markers that are pointed upward, as depicted in Figure 10. The HyperCube position of reference was therefore measured in real time with a sub-millimetric precision.

### 4.1. Calibration

In this work, we consider that the HyperCube sensor can move in a range of [−50 mm; +50 mm] in both *X* and *Y* directions. Given *Z_LED_* = 300 mm, one can assume that the angles are small enough to write from Equation (9) *S_φ_* = −*tan*(*φ*) ⋍ −*φ* for azimuth and from Equation (11) *S_ψ_* = −*tan*(*ψ*) ⋍ −*ψ* for elevation. As shown in the experimental setup in Figure 10, we theoretically have $S_{\varphi }=-\tan\varphi =\frac{X_{\text{LED}}}{Z_{\text{LED}}}$ and $S_{\psi }=-\tan\psi =\frac{Y_{\text{LED}}}{Z_{\text{LED}}}$, where *X_LED_, Y_LED_* and *Z_LED_* are the relative position of the IR LED to the HyperCube sensor. The positions of reference *X_LED_, Y_LED_* and *Z_LED_* are provided by the tracking system VICON.

**Figure 11:**
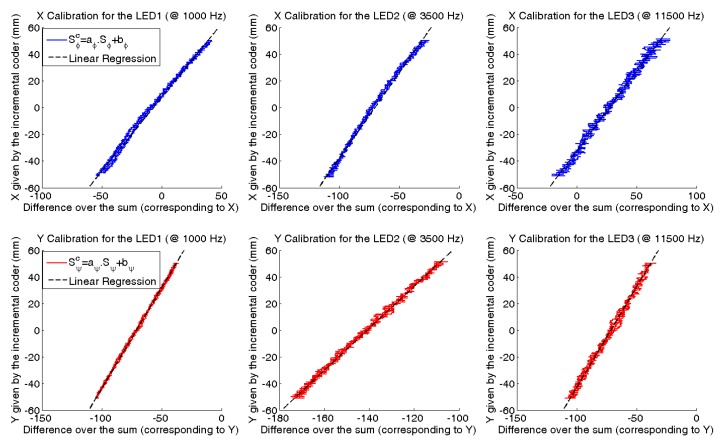
*X_LED_* and *Y_LED_* are measured by an incremental encoder for each direction and are plotted as a function of *S_φ_* and *S_ψ_*. Linear regressions used to determine *a_φ_, a_ψ_, b_φ_* and *b_ψ_* are also plotted. We note that the curves are linear on the range displayed.

The calibration consists of adjusting the HyperCube outputs *S_φ_* and *S_ψ_* to the ratios $\frac{X_{\text{LED}}}{Z_{\text{LED}}}$ and $\frac{Y_{\text{LED}}}{Z_{\text{LED}}}$. To this aim, the calibration curves shown in Figure 11 are plotted. We note that the HyperCube outputs are linear in the observed range [−50 mm; +50 mm]. Linear regression computes the slope (*a_φ_, a_ψ_*) and the offset (*b_φ_, b_ψ_*) by fitting each curve with a linear model. These coefficients calibrate the HyperCube outputs $S_{\varphi }^{c}$ and $S_{\psi }^{c}$, such that: $S_{\varphi }^{c}=a_{\varphi }.S_{\varphi }+b_{\varphi }=\frac{X_{\text{LED}}}{Z_{\text{LED}}}=-\varphi$ and $S_{\psi }^{c}=a_{\psi }.S_{\psi }+b_{\psi }=\frac{X_{\text{LED}}}{Z_{\text{LED}}}=-\psi$. Figure 11 presents the calibration results for each IR LED in both the *X* and *Y* directions. The sensor's outputs are linear in the range [−50 mm; +50 mm] with respect to the origin.

The calibration validation protocol takes *Z_LED_* = 300 mm. The estimated positions *X̂_LED_* and *Ŷ_LED_* of the IR LED were compared to the references *X_LED_* and *Y_LED_* provided by the VICON system.

### 4.2. Height Estimation

Form the experimental setup described in Figure 10 and given the signal outputs *S_ψ_* and *S_φ_* and two IR LEDs, one can assess the height *Z_LED_* between the sensor and two IR LEDs A and B.

**Figure 12:**
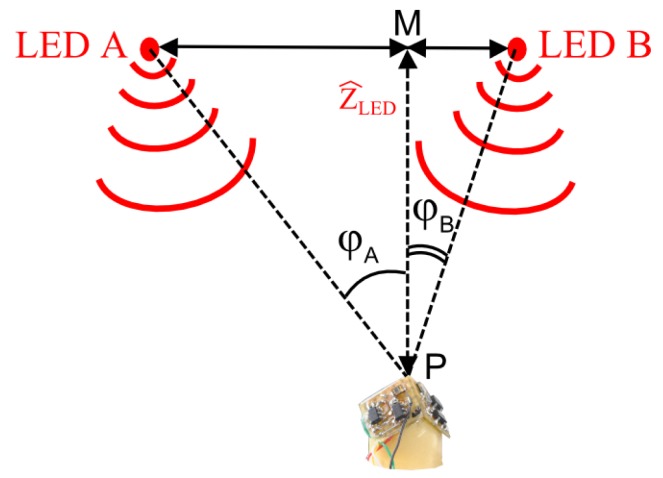
Height estimation description while HyperCube assesses the azimuthal angles *φ_A_* and *φ_B_* thanks to the signal output *S_φ_*.

The assessed height *Ẑ_LED_* is given *by* the relation: ${\hat{Z}}_{\text{LED}}=\frac{AB}{\tan\left(\varphi_{A}\right)+\tan\left(\varphi_{B}\right)}$ where AB is the distance between the LEDs A and B. *φ_A_* and *φ_B_* are the azimuthal angles given by HyperCube for the LEDs A and B. In the configuration described in Figure 12, *Y_LED_* = 0 (*i.e.*, ABPis orthogonal to the horizontal plane that intersects HyperCube).

## 5. Experimental Position Estimation in 2D

In this section, we used a single IR LED and HyperCube for each experiment.

**Figure 13:**
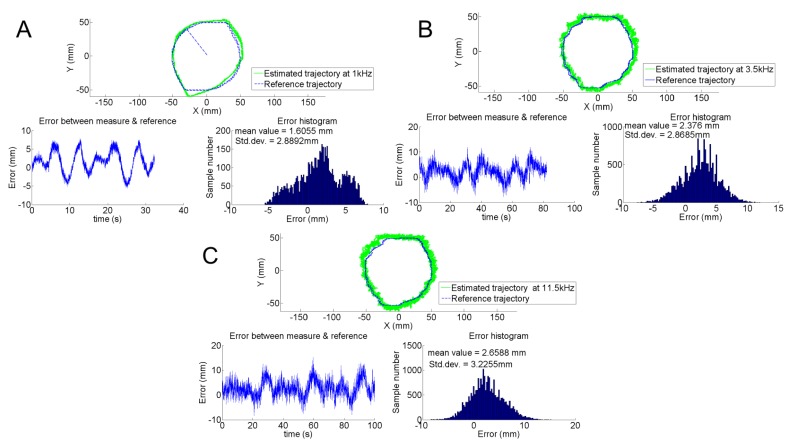
(**A**) Position estimation for a circular trajectory with a radius of 50 mm in the XY plane. Note that the absolute value of the position error is lower than 1 cm and that the standard deviation is about 3 cm; We also note that the noise is more important for the experiments at 3.5 kHz (**B**) and 11.5 kHz (**C**).

We compared the estimation to HyperCube positions assessed by the tracking system for three different modulation frequencies. For the IR LED that flickers at 1 kHz, Figure 13A shows the plots of the reference trajectory as a dashed line and the trajectory depicted by HyperCube as a solid line. The trajectory is a circular path with a radius of 50 mm in the XY plane with *Z_LED_* = 300 mm. The distance between reference and estimated trajectories was also plotted. The histogram of the error gives a mean value *μ* = 1.6 mm and a standard deviation *σ* = 2.8 mm. We noted that the noise at 1 kHz for the demodulation was lower than the noise at 3.5 kHz and 11.5 kHz, as shown in Figure 13B,C. The higher levels of noise at 3.5 kHz and 11.5 kHz than the one at 1 kHz are due to the use of different operational amplifiers (TI OPA4322 and TIOPA4342) for the analog demodulation. Figure 13A-C highlight that the HyperCube sensor provides useful measurements to accurately estimate the dynamic relative position of an IR LED in the XY plane.

To reduce the measurement noise, the digital signal outputs given by the HyperCube sensor were filtered by a Kalman filter. The system is described in a state space form as follows:

(16) $$x_{k+1}=Fx_{k}+v_{k},$$

(17) $$y_{k}=Cx_{k}+w_{k}\;\text{with}\;k\in \mathbb{N}$$

where the state *x_k_* = [*X_k_ Ẋ_k_*]*^T^* describes the position *X_k_* and the velocity *Ẋ_k_* at the time instant *t_k_*. *w_k_* and *v_k_* are the state and measurement white noise with known and constant covariance matrices *W* and *V*, respectively. They are supposed to be mutually uncorrelated. $F=\left[\begin{array}{cc} 1 & T_{e}\\ 0 & 1 \end{array}\right]$ is the state transition matrix, *C* = [1 0] the observation matrix. In practice, we choose the covariance matrices as follows $V=\sigma_{\text{meas}}^{2}\left[\begin{array}{cc} 1 & 0\\ 0 & 1 \end{array}\right]$ and $W=\sigma_{\text{proc}}^{2}\left[\begin{array}{cc} 1 & 0\\ 0 & 1 \end{array}\right]$ where *σ_meas_* and *σ_proc_* are the standard deviations of the measurement noise and the state noise, respectively.

A linear Kalman filter was also implemented for the state estimation along the *Y* direction.

### 5.1. Experimental Height Estimation

**Figure 14:**
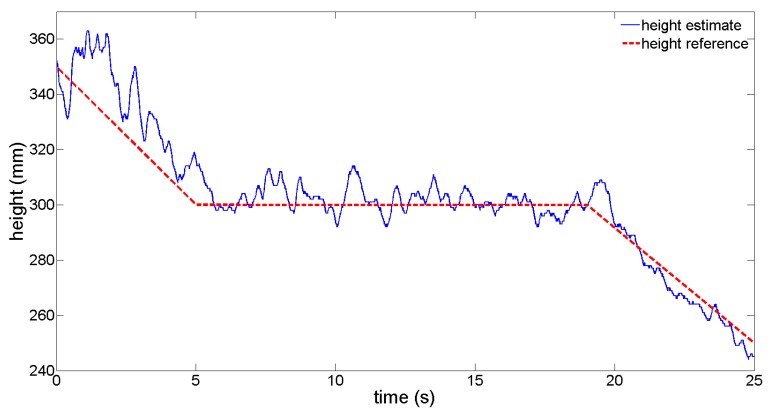
Mean of the height estimate for three pairs of IR LEDs (see Equation (18)) while the position of the HyperCube sensor is fixed. Note that the height estimation is more accurate at 300 mm, since this was the height of calibration.

As described in Section 4.2, the height between the HyperCube sensor and a pair of IR LEDs was estimated. Thanks to the angular measurements using three flickering IR LEDs, one can reduce the measurement noise. The height estimation precision is therefore increased. The mean of the height estimate *Ẑ̅* is written as:

(18) $$\bar{\hat{Z}}=\frac{{\hat{Z}}_{\text{LED}1,2}+{\hat{Z}}_{\text{LED}2,3}+{\hat{Z}}_{\text{LED}1,3}}{3}$$

where *Ẑ_LED_*_1,2_, *Ẑ_LED_*_2,3_ and *Ẑ_LED_*_1,3_ are the height estimated by the pairs (1,2), (2,3) and (1,3), respectively. The height of the IR LEDS was modified, while the position of the HyperCube sensor was fixed in the XY plane. Using each pair of IR LEDs, the height estimation was computed on-line. From Figure 14, the height estimation as a solid line is precise around 300 mm with respect to the reference height as a dashed line.

**Figure 15:**
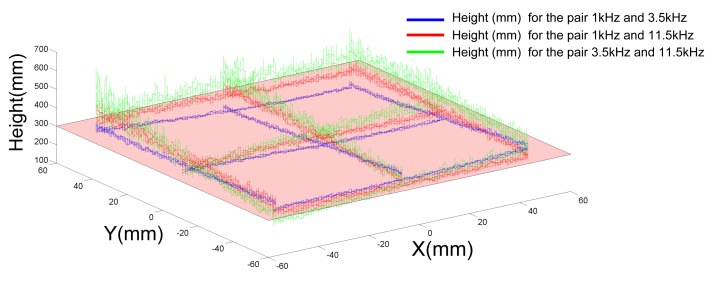
Height estimation for three pairs of LEDs and with a 10-cm shift in the *X* and *Y* directions. Note that a small coupling appears on the height estimation with respect to the position of HyperCube in the XY plane.

In Figure 15, for different pairs of IR LEDs, we plotted the evolution of the height estimation while the sensor was moving relatively to the center of the pair of IR LEDs. We note from Figure 15 that the height estimation depends on the position (*X_LED_, Y_LED_*) of the sensor. The estimation error can vary up to 50 mm.

The estimation remains better with the pair of IR LEDs (1 kHz and 3.5 kHz) than with the others.

### 5.2. IR LED Position Tracking in 2D

**Figure 16:**
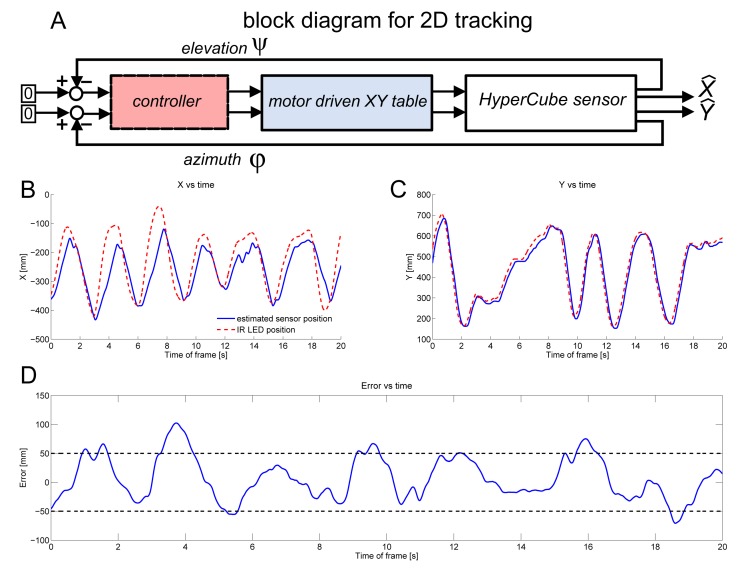
(**A**) Block diagram of the IR LED position tracking system characterized by moving by hand one IR LED (flickering frequency of 1 kHz) placed 30 cm above HyperCube; (**B**) experimental recording of the position tracking along the *X* direction *versus* time. The DC motor dynamics in charge of moving HyperCube along the *X* axis was a bit too slow to follow faithfully the quick variations of the reference input signal; (**C**) experimental recording of the position tracking along the *Y* direction *versus* time; (**D**) error of the IR LED position tracking. Note that the absolute value of the position error never exceeds 10 cm.

In this section, HyperCube measurements, which consist of azimuth *φ* and elevation *ψ*, feed the controller that aims at tracking a moving IR LED in the plane. A Smith predictor with proportional and integral actions was designed and implemented to ensure the good stability of the linear displacement of HyperCube despite the time lag inherent to the visual processing algorithm. Figure 16A shows the block diagram of the closed loop system with the assessed angles *φ* and *ψ*. In Figure 16B,C, estimated positions versus time are plotted. The tracking system gives the reference trajectory.

As shown in Figure 16B,C, the position tracking was accurate. The tracking error plotted in the Figure 16D shows that the error ranges within ±5 cm. Nevertheless, one can note in Figure 16B that there is an error between the estimated and the reference values due to a saturation of the motor of the *X* axis. Thanks to a fast motor for controlling the *Y* axis, HyperCube was able to follow faithfully the moving pattern. In Figure 16C, the HyperCube sensor perfectly tracks the moving IR LED along the *Y* axis.

## 6. Conclusions

We designed and presented the HyperCube sensor. It is a cubic, miniature and low-cost sensing device able to track active markers without any optics. The system discussed has the following key features:

1. It provides azimuth and elevation and is able to assess the angular position of three blinking IR LEDs at specific frequencies: 1 kHz, 3.5 kHz and 11.5 kHz.
2. The estimation is achieved by a computationally-inexpensive signal processing of the low-amplitude and high-frequency signal transmitted by the three photodiodes placed on each side of the cube.
3. Experimental results showed that for the position estimation and the position tracking in 2D, this miniature device is very efficient. It also features promising results in terms of distance estimation, as well.

Nevertheless, the prototype developed reveals some limitations with respect to the range of pose estimation, but it is well adapted for proximal localization (up to 30 cm). In addition, the size of the demodulation electronic board could be easily reduced. As a conclusion, the HyperCube sensor is a promising sensing device for proximal localization applications and could equip UAVs or MAVs. The short-term outlook consists of improving the range of sensitivity of HyperCube using silicon photodiodes, which are specifically designed for precision photometry and feature a high sensitivity, a high-speed response and low noise.
